# Retrospective Analysis of Laparoscopic Varicocelectomy in Pediatric Patients: Impact of Lymphatic-Sparing Techniques and Methylene Blue on Outcomes—A Series of Cases

**DOI:** 10.3390/jcm14113814

**Published:** 2025-05-29

**Authors:** Arzu Canmemis, Meltem Caglar, Cigdem Ulukaya Durakbasa

**Affiliations:** 1 Division of Pediatric Urology, Department of Pediatric Surgery, Goztepe Prof. Dr. Suleyman Yalcin City Hospital, 34722 Istanbul, Türkiye; 2 Department of Pediatric Surgery, Goztepe Prof. Dr. Suleyman Yalcin City Hospital, 34722 Istanbul, Türkiye; 3 Department of Pediatric Surgery Istanbul, Faculty of Medicine, Istanbul Medeniyet University, 34730 Istanbul, Türkiye

**Keywords:** varicocele, lymphatic sparing, methylene blue, testicular calcification, complications

## Abstract

**Objective**: This study aimed to evaluate the outcomes and complications of laparoscopic varicocelectomy (LV) in pediatric and adolescent patients, comparing lymphatic-sparing (LS) and non-lymphatic-sparing (NLS) techniques, with a particular focus on the postoperative effects of methylene blue dye. **Methods**: A retrospective review was conducted for patients with Grade 3 left-sided varicocele who underwent LV between 2010 and 2023. Patients were grouped according to whether lymphatic-sparing techniques were used. Testicular volumes were measured pre- and postoperatively by ultrasonography. Surgical complications including hydrocele, recurrence, and intratesticular calcification were recorded. Statistical comparisons were made between the two groups. **Results**: A total of 21 patients with a median age of 15 years were included. LS suvrgery was performed in seven patients (33.3%), and arterial sparing in four (19%). Methylene blue was used to identify lymphatic vessels in the LS group. Postoperative hydrocele was observed in three patients (14.3%) and occurred equally in both groups. However, testicular calcification was detected only in the LS group and was significantly more common compared to the NLS group (*p* = 0.002). Recurrence rates were not significantly different between groups (*p* = 0.694). No cases of testicular atrophy were observed. The median follow-up duration was 6 years in the NLS group and 3 years in the LS group (*p* = 0.026). **Conclusions**: Lymphatic-sparing laparoscopic varicocelectomy appears effective in preserving testicular volume, but the use of intratesticular methylene blue is associated with a higher incidence of calcification. These findings highlight the need for caution and further long-term evaluation of vital dyes in pediatric varicocelectomy.

## 1. Introduction

Varicocele is an abnormal enlargement of the pampiniform plexus due to venous reflux [1]. Its prevalence is approximately 15% in children and adolescents, with most cases occurring on the left side and about 10% presenting bilaterally [2]. Testicular asymmetry is generally considered a potential indicator of long-term subfertility and is thus used as a criterion for treating adolescent varicocele. According to current guidelines, surgery is strongly recommended in cases of persistent small testis (>2 mL or 20% asymmetry), while the level of recommendation is weaker for other indications [3].

Surgical treatment involves ligation of the internal spermatic veins, with options including Ivanissevich’s inguinal approach and the high retroperitoneal Palomo procedure [4]. Other options for suprainguinal ligation include inguinal (or subinguinal) microscopic and laparoscopic techniques [4,5,6,7]. In adults, microsurgical varicocelectomy is considered the most effective and safe technique [6]. Complication rates vary among different surgical techniques and there is no gold standard technique for children and adolescents. The laparoscopic Palomo technique is recognized as a safe and effective approach for treating varicocele in the pediatric population, with reported success rates exceeding 95% [8].

Common complications include testicular edema and hydrocele formation [9]. Postoperative hydrocele formation represents the most common complication following adolescent varicocelectomy. Reported prevalence ranges from 0% to 30%, and it is widely considered that the surgical technique employed plays a significant role in influencing this risk [4,10,11,12]. In a meta-analysis, postoperative recurrence rates were reported to range from 0% to 12%, while testicular atrophy rates ranged from 0% to 2.9% [1].

Intraoperative lymphography has been shown to facilitate the preservation of lymphatic vessels during surgery, and various techniques have been described to enable lymphatic mapping intraoperatively. In 2001, Oswald et al. introduced the use of intrascrotal isosulfan blue injection as a method to enhance the visualization of lymphatic structures requiring preservation [12]. Since then, alternative approaches have emerged, involving different vital dyes—such as methylene blue and, more recently, indocyanine green—as well as variations in injection sites, including intratesticular and intrascrotal administration [13,14]. Lymphatic-sparing varicocelectomy is preferred to prevent hydrocele formation, testicular hypertrophy, and to maintain better testicular function, and recommended by the current EUA guidelines [3,7,9]. A recent systematic review and meta-analysis by Tandon et al. highlighted the varying rates of recurrence and complications among different surgical techniques for pediatric varicocelectomy. Their study emphasizes the importance of technique selection, noting that lymphatic-sparing laparoscopic surgery presents the lowest recurrence and complication rates compared to other methods [1].

Intratesticular injection appears to be the most effective method for lymphatic mapping, achieving a 100% success rate in identifying lymphatic vessels, compared to approximately 80% with intrascrotal injection [14,15,16]. Nevertheless, concerns have been raised regarding the potential for testicular alterations following intratesticular dye administration. Despite these concerns, studies specifically investigating this issue remain limited in the literature [13,15].

This study aims to evaluate the long-term outcomes and complications of laparoscopic varicocelectomy with and without lymphatic-sparing in pediatric patients at a single center.

## 2. Materials and Methods

This study was a retrospective analysis conducted on patients diagnosed with Grade 3 left-sided varicocele. The patients were divided into two groups: those who underwent lymphatic-sparing varicocelectomy and those without lymphatic sparing.

Patients who underwent laparoscopic varicocelectomy and whose clinical data were accessible through a retrospective review of hospital records were included in the study. Patients were excluded if their retrospective records were incomplete or insufficient, if the surgical technique could not be clearly identified, if they were lost to follow-up, or if they had undergone inguinal varicocelectomy rather than laparoscopic varicocelectomy.

This study was conducted in accordance with the principles outlined in the Declaration of Helsinki, as revised in 2013. Written informed consent was obtained from all patients or their guardians prior to surgery. Ethical approval was obtained from the Istanbul Medipol University Ethics Committee (992/2024). Written informed consent was obtained from all patients or their guardians prior to surgery.

Patient demographics, intraoperative variables, and postoperative outcomes were collected from medical records. All patients underwent preoperative Doppler ultrasonography. Preoperative and postoperative testicular volumes for both right and left testicles (RTV and LTV, respectively) were measured using ultrasonography. Testicular volume was measured using ultrasound with the formula: 0.52 × length × width × height [17]. The differences between RTV and LTV preoperatively and postoperatively were recorded, alongside volume change percentages to evaluate the impact of the surgery. Complications were categorized and graded according to the Clavien-Madadi classification, with data on hydrocele, calcification, and recurrence specifically noted [18]. All patients were followed up at 1, 6, and 12 months postoperatively, and then annually. Scrotal USG was performed during each visit to evaluate testicular volumes. In this study, the latest follow-up data were used for analysis.

The indications for varicocelectomy included high-grade varicocele (Grade II or III according to the Dubin and Amelar clinical classification), left testicular hypotrophy defined as a volume differential of 15% or more between testicles, or associated symptoms such as pain or discomfort in the left testicle [19].

**Figure 1:**
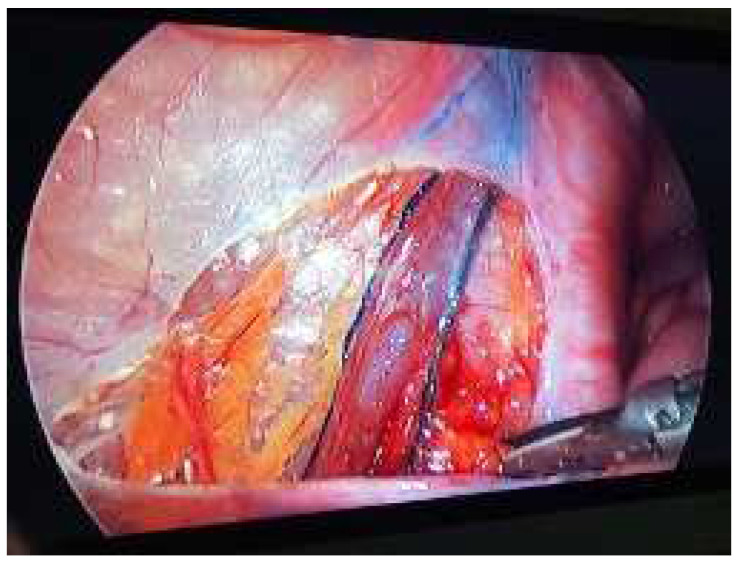
Intraoperative appearance of the lymphatics stained with methylene blue.

Lymphatic sparing was performed using methylene blue to visualize and protect the lymphatic vessels. Surgical approaches and technique selection were based on the surgeon’s preference. All surgeries were performed under general anesthesia with the patient in the supine and Trendelenburg positions. A 5 to 10 mm umbilical trocar for the 0° optic was consistently used in laparoscopy, with two additional 5 mm working trocars placed in a triangulated configuration. The posterior peritoneum overlying the internal spermatic vessels (ISVs) was opened with a monopolar hook, approximately 2 cm in length, at a distance of 3–4 cm from the internal inguinal ring. A window was created behind the ISV, and the vessels were sealed divided according to the preoperative surgical plan. In the classic Palomo technique, the entire bundle was transected with a sealing device. For the artery-sparing technique, after isolating the artery, it was preserved while the remaining vessels were transected with a sealing device. In the lymphatic-sparing group, after the entire bundle was exposed, 0.5–0.6 mL of methylene blue (50 mg/10 mL) was injected intratesticularly using a 26-gauge insulin needle. Approximately five minutes later, the lymphatic vessels became visible due to methylene blue staining. The identified lymphatic vessels were separated from the artery and vein. Only vascular structures were transected using a sealing device (Figure 1). Artery-sparing varicocelectomy was performed with the assistance of laparoscopic magnification.

For follow-up, patients were initially seen one week postoperatively for a surgical wound check. Subsequent clinical evaluations were conducted at 1, 6, and 12 months post-surgery, followed by annual assessments (t0: preoperative, t1: follow-up at first month, t2: follow-up at 6 months, and t3: follow-up at 12 months). These visits focused on checking for varicocele persistence, recurrence, testicular volume, catch up rates of growth of the left testicle or the emergence of a new hydrocele. Testicular ultrasound was repeated whenever feasible.

## 3. Statistical Analysis

Statistical analyses were performed to compare demographic and clinical characteristics between the lymphatic-sparing and non-lymphatic sparing groups. Continuous variables were tested for normality using the Shapiro–Wilk test and are presented as medians with interquartile ranges (IQRs) when the variables did not fit normality criteria. For comparisons, the Mann–Whitney U test was used for continuous variables, and Fisher’s exact test was used for categorical data. A dependent T-test analysis was performed to compare preoperative and postoperative RTV, LTV, and RTV-to-LTV volume differences. A Sankey diagram was generated to illustrate the relationship between complications and surgical approaches. Jamovi software version 2.4 was used [20]. Statistical significance was set at *p* < 0.05.

## 4. Results

**Figure 2:**
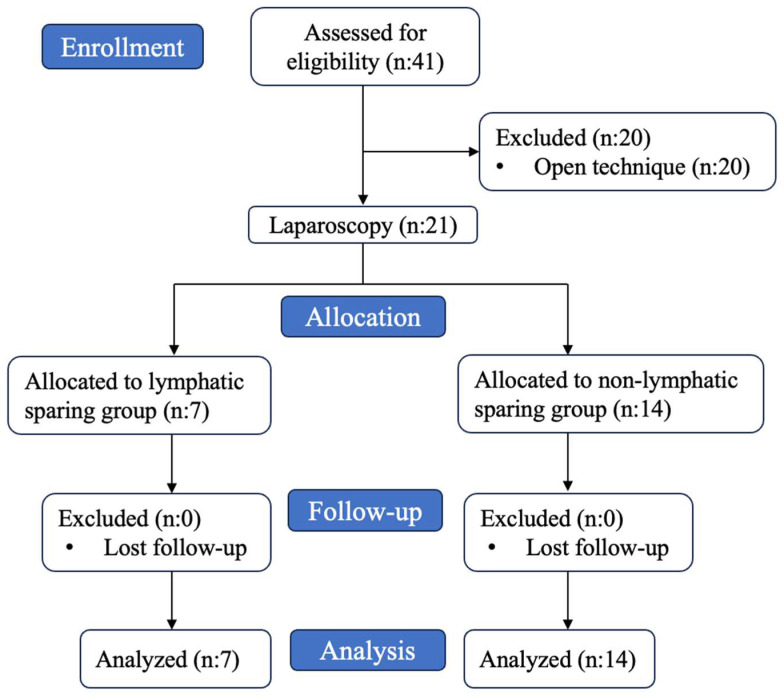
Flow chart of included patients.

All procedures were performed by two experienced surgeons under general anesthesia. A total of 41 patients underwent varicocelectomy in our clinic between 2010 and 2023. Of these, 20 patients underwent inguinal varicocelectomy and are excluded from further analysis. Laparoscopic varicocelectomy was performed in 21 patients. A flow chart of patient selection was given in Figure 2.

A total of 21 patients were included. All patients had left-sided Grade 3 varicoceles. The median age was 15 (13.5–17) years. Lymphatic sparing by way of methylene blue injection was performed in 33.3% of the patients (n = 7), while arterial sparing was applied in 19% (n = 4), all after the year 2021. The remaining 10 (47.6%) patients underwent classical Palomo varicocelectomy. Methylene blue injection enabled a clear visualization of lymphatic vessels in five patients but it was less distinct in two patients.

The median preoperative right testicular volume (RTV) was 6.8 mL (3.48–7.5), and the median left testicular volume (LTV) was 5.98 mL (3.33; 7.6). The median preoperative RTV-to-LTV difference was 0.23 mL (−0.685–2.15). In the postoperative period, the median RTV was 7.04 mL (3.87–8.05), and the LTV was 5.96 mL (4.62–8.48). The median postoperative RTV-to-LTV difference was found to be 0.05 mL (−0.717–1.21). The median change in RTV was 38% (5.74–129), and the median change in LTV was 72.5% (−21.9–145).

Among the complications, hydrocele was observed in three patients (14.3%), calcification in four patients (19%), and recurrence in four patients (19%). The distribution of surgical complications according to the Clavien-Madadi classification was as follows: Grade 1 complications were observed in six patients (29%), and Grade 3B complications in three patients (14.3%). No intraoperative complications or atrophy during the follow-up period were observed in any of the patients (Table 1).

**Figure 3:**
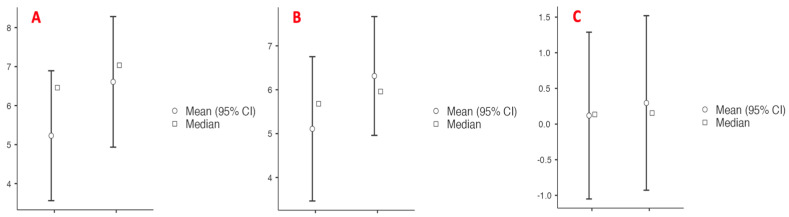
Dependent T-test analysis graph. (**A**) Preoperative RTV vs. Postoperative RTV. (**B**) Preoperative LTV vs. Postoperative LTV. (**C**) Preoperative RTV-to-LTV vs. Postoperative RTV-to-LTV.

**Figure 4:**
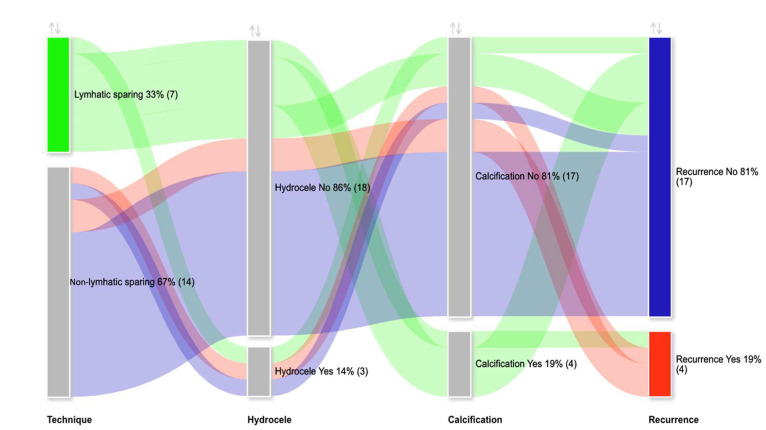
Sankey diagram of relationship between complications and surgical approach.

Patients were analyzed in two groups as non-lymphatic sparing (n = 14) and lymphatic sparing (n = 7). Arterial sparing was carried out in four (19%) of the non-lymphatic sparing group and none in the lymphatic sparing group, but this was not statistically significant (*p* = 0.131). Preoperative RTV and LTV were similar between the groups (RTV: *p* = 1.000; LTV: *p* = 0.966). Postoperatively, the median RTV increased by 38% in the non-lymphatic sparing group and 68.8% in the lymphatic sparing group (*p* = 0.933). The median LTV increased by 34.6% in the non-lymphatic sparing group and 110% in the lymphatic sparing group (*p* = 0.683) (Figure 3). Complications included hydrocele development in three (14.3%) patients in each group (*p* = 1.000). One patient from each group underwent surgery due to hydrocele while one patient had minimal hydrocele detected on ultrasound, which resolved after a one-year follow-up. Intratesticular calcification development was significantly more common in the lymphatic-sparing group (51.7% vs. 0%; *p* = 0.002). The recurrence rates were comparable between the groups (21.4% vs. 14.3%; *p* = 0.694). The median follow-up duration was significantly longer in the non-lymphatic sparing group, at 5.5 years (2.6; 7), compared to 3 years (2; 3) in the lymphatic sparing group (*p* = 0.320). The postoperative complications according to Clavien-Madadi classification were distributed without significant difference (*p* = 0.343) (Table 2 and Figure 4).

A Sankey diagram was generated to facilitate the comparison of complications across different surgical approaches (Figure 4).

When the characteristics of patients who underwent arterial sparing are compared to those who did not, there was no statistically significant difference in terms of the demographic data, the clinical outcomes, and the complications (Table 3).

**Figure 5:**
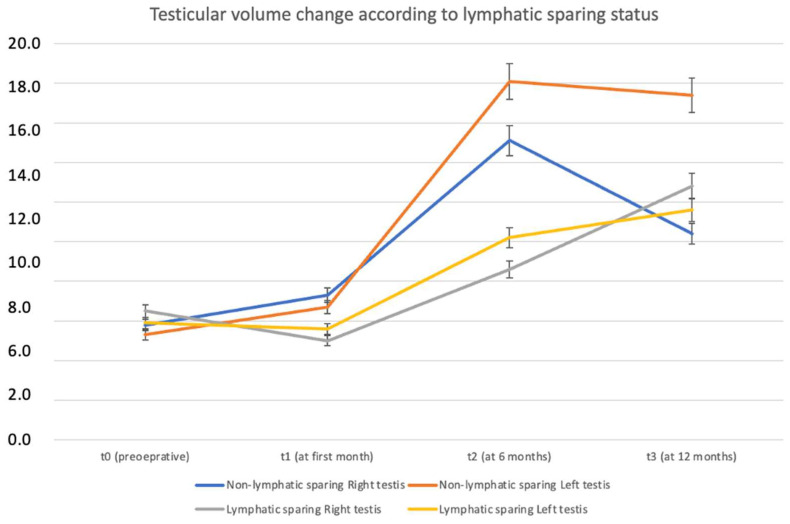
Testicular volume change by time according to lymphatic sparing status.

**Figure 6:**
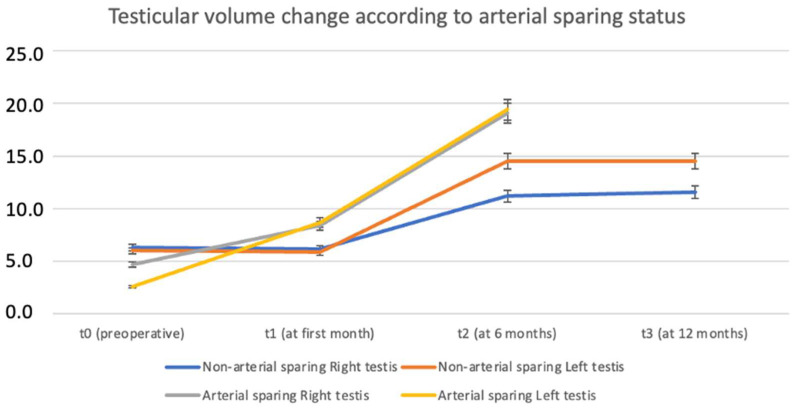
Testicular volume change by time according to arterial sparing status.

Patients who underwent lymphatic-sparing procedures exhibited a more consistent and sustained increase in testicular volume over time, particularly at t2 and t3 time points. This finding suggests that lymphatic sparing may contribute positively to postoperative testicular growth and preservation (Figure 5). Similarly, the analysis of testicular volume change based on arterial sparing status revealed a notably greater increase in volume among patients who underwent arterial-sparing procedures, particularly evident at 6 and 12 months postoperatively (t2 and t3). These findings indicate a potential benefit of arterial preservation in promoting long-term testicular growth and function (Figure 6).

## 5. Discussion

The main indications for varicocele surgery are pain and fertility issues. Second- and third-degree varicoceles can lead to decreased testicular volume and impact hormone levels (FSH, LH, inhibin B) [21,22,23]. Numerous studies have compared different surgical methods [24,25]. The Palomo technique was first described in 1949 and involves ligating all dilated testicular vessels at the level of the internal ring [26]. Laparoscopic outcomes of the Palomo technique were first reported in 1994, and it has since become widely used [27].

One of the common complications of varicocele surgery is postoperative hydrocele development, which is believed to result from lymphatic obstruction. Occurrence of this complication is reported at a quite variable rate in different studies. It occurred in 13.8% of patients who underwent laparoscopic Palomo procedure, while none was observed in those who underwent lymphatic-sparing surgery [28,29]. However, some studies reported hydrocele development rates as high as 40% in laparoscopic repairs [13,30,31]. Other studies showed a reduction in hydrocele development rates with lymphatic-sparing techniques [10,11]. This variability could be due to different criteria used to diagnose postoperative hydrocele, and a previously published study may help establish consensus on this issue [32]. Our results showed that hydroceles developed in three patients (14.3%) with no significant difference between non-lymphatic sparing and lymphatic-sparing groups. A systematic review by Tandon et al. supports our findings, demonstrating that lymphatic-sparing laparoscopic varicocelectomy significantly reduces postoperative hydrocele formation and recurrence rates compared to conventional techniques. However, they noted the importance of long-term follow-up due to potential complications related to different surgical approaches [1].

In our study, no cases of testicular atrophy were observed. This may be attributed to high ligation of the spermatic vessels at the retroperitoneal level, which helps preserve distal testicular blood flow. The literature suggests that transection of the vessels approximately 3–4 cm proximal to the internal inguinal ring may protect collateral circulation and thereby maintain adequate testicular perfusion [33]. Our findings are consistent with this approach and align with the results reported in a recent meta-analysis [1].

In the study by Hung et al., subdartos injection of methylene blue during lymphatic-sparing varicocelectomy resulted in new ultrasonographic testicular lesions in 22.2% of patients. These changes included hypodense and hyperechogenic areas as well as microcalcifications, with most persisting for at least two years. Similarly, in our study, testicular calcifications were observed exclusively in patients who received intratesticular methylene blue injection, and the incidence was significantly higher compared to those without dye use (51.7% vs. 0%, *p* = 0.002). While both studies confirm the utility of methylene blue in lymphatic mapping, they also highlight its potential adverse effects on testicular tissue. Hung et al. underscored the need for further evaluation of these ultrasonographic findings through hormonal and semen analyses, as their functional implications remain unclear [13]. Similarly, the long-term clinical significance of calcifications observed in our cohort is yet to be determined. These results underscore the importance of cautious use of methylene blue and the necessity for prospective studies involving long-term follow-up and investigation of safer alternative agents.

Lymphatic-sparing surgery was initially defined by M. Goldstein et al. during microsurgical varicocelectomy and has gained significant popularity since the 2000s [5]. The goal of lymphatic preservation is not only to reduce hydrocele formation, but also to decrease testicular edema, which can cause pseudo-enlargement and impair testicular function [10]. A meta-analysis reported an average catch-up growth rate of 76.4% [34]. In our study, left testicular growth was greater in patients who underwent lymphatic-sparing surgery, though not statistically significant, likely due to growth without edema development. Although the differences did not reach statistical significance, our findings suggest that lymphatic-sparing varicocelectomy may contribute to sustained postoperative testicular growth, particularly evident at mid- and long-term follow-up intervals. Serial ultrasonographic measurements demonstrated a greater median increase in left testicular volume among patients who underwent lymphatic preservation.

The first use of vital dyes to visualize lymphatic vessels in varicocele surgery involved intrascrotal methylene blue injection by Tan et al. [35]. Mapping techniques include dyes like methylene blue, isosulfan blue, and indocyanine green [14,15,27,28]. Injecting isosulfan blue between the tunica vaginalis and tunica albuginea results in successful lymphatic mapping in 70–90% of cases [15]. Esposito et al. achieved 100% mapping by injecting 0.5 mL of isosulfan blue intraparenchymally [14]. Methylene blue’s mapping success rate for lymphatics ranges from 93 to 100% across various techniques, and its safety is well documented [36,37]. In our study, lymphatic vessels were clearly visualized in five patients (71%) and less distinctly in two (29%) patients. Our lower mapping rates may be attributed to the use of dye volumes smaller than the 1–2 mL typically reported in the literature.

Vital dyes allow for clear visualization of lymphatic vessels, but long-term data on their effects on the testis are limited. A few studies have linked vital dyes to testicular calcification and de novo testicular changes, even when injected outside the parenchyma [13,38,39]. Studies using indocyanine green also reported a 4% incidence of postoperative calcifications in the testis, though some resolved during follow-up [15]. In our series, testicular calcifications were observed only in patients who underwent lymphatic mapping with methylene blue. Methylene blue was chosen for lymphatic mapping because it is easily accessible, affordable, has a low risk of anaphylaxis, and has been proven safe in the literature [40]. Although there is no defined gold standard surgical method for pediatric varicocele, the current EUA guidelines recommend lymphatic-sparing surgery with dye assistance [3]. However, long-term data on intratesticular injection outcomes, particularly with respect to subsequent testicular calcifications, remain a concern. In a study with a mean follow-up of approximately 23 months, Esposito et al. concluded that calcification development was not clinically significant, as patients were asymptomatic, and serum tumor markers were normal in all cases [14]. They hypothesized that the calcifications might result from injection volume (2 mL) causing compartment syndrome in the testis, or needle size may play a role. Although our injection volume was no more than 0.6 mL and we used a 26-gauge needle, future hormonal or semen analyses will be needed to determine if testicular function is maintained in patients with calcifications. Prospective large-scale studies are needed to clarify the clinical significance of these findings.

In the suprainguinal approach, preserving the testicular artery did not provide additional benefits for testicular growth, and higher recurrence rates were reported [41]. In the published studies, recurrence rates for both open and laparoscopic surgeries vary, but our results were consistent with the literature [42,43]. No recurrences were observed in patients who underwent artery-sparing varicocelectomy; however, no statistically significant difference was found compared to the group without artery-sparing varicocelectomy. We attribute our high recurrence rates to the inclusion of both clinically detected and low-grade recurrences identified on ultrasound.

The follow-up duration differed between groups, as we started performing dye-assisted lymphatic-sparing surgery only after 2021. However, macroscopic calcifications were observed only in patients treated with methylene blue, prompting us to reconsider our surgical technique.

The study’s strengths lie in its execution by experienced surgeons within a single center, which ensures procedural consistency and enhances the reliability of the results. The relatively long follow-up period allowed for comprehensive assessment of both the surgical outcomes and potential long-term complications. By comparing lymphatic-sparing and non-lymphatic-sparing laparoscopic Palomo techniques, the study provides valuable insights into the efficacy and complication profiles of different surgical approaches. The intraoperative use of methylene blue to visualize and preserve lymphatic vessels adds a distinctive and innovative aspect to the research. Furthermore, the objective evaluation of testicular volume changes using ultrasonography contributes to the robustness and accuracy of the findings.

The limitations of this study include its retrospective design and small sample size. The hormone levels and future outcomes of patients with calcifications warrant further investigation.

## 6. Conclusions

Varicocele contributes to male infertility by negatively impacting testicular volume and sperm parameters. In our study, we observed that laparoscopic varicocelectomy with lymphatic preservation was associated with similar hydrocele rates but an increased testicular calcification risk. Although lymphatic preservation is known to reduce hydrocele risk, long-term data on the impact of vital dyes like methylene blue on testicular parenchyma are limited. Our findings suggest caution when performing lymphatic-sparing surgery with methylene blue in pediatric and adolescent patients. Long-term follow-up and larger sample sizes in future studies will be valuable for assessing the safety and long-term effects of this technique.

## Figures and Tables

**Table 1:** Characteristics of all patients.

	n (%); Median (Q1–Q3)
Age	15 (13.5–17)
Side (left)	21 (100%)
Grade 3 varicocele	21 (100%)
Lymphatic sparing	7 (33.3%)
Arterial sparing	4 (19%)
Preoperative RTV (mL)	6.8 (3.48–7.5)
Preoperative LTV (mL)	5.98 (3.33–7.6)
Preoperative RTV-to-LTV (mL)	0.23 (−0.685–2.15)
Postoperative RTV (mL)	7.04 (3.87–8.05)
Postoperative LTV (mL)	5.96 (4.62–8.48)
Postoperative RTV-to-LTV (mL)	0.05 (−0.717–1.21)
Change in RTV (%)	38 (5.74–129)
Change in LTV (%)	72.5 (−21.9–145)
Hydrocele	3 (14.3%)
Calcification	4 (19%)
Recurrence	4 (19%)
Clavien-Madadi Classification	
1	6 (29%)
3B	3 (14.3%)

RTV: right testes volume; LTV: left testes volume; RTV-to-LTV: difference in right and left testicular volume.

**Table 2:** Comparison according to lymphatic sparing surgery.

	Non-Lymphatic Sparing (n = 14)	Lymphatic Sparing (n = 7)	*p*-Value
Age	15.5 (15–17)	13 (11.5–16)	0.247
Side (left)	14 (100%)	7 (100%)	1.000
Grade 3 varicocele	14 (100%)	7 (100%)	1.000
Arterial sparing	4 (28.5%)	0 (0%)	0.116
Preoperative RTV (mL)	6.8 (2.73–7.56)	6.72 (2.73–7.36)	1.000
Preoperative LTV (mL)	6.03 (3.53–7.32)	5.20 (2.60–8.36)	0.966
Preoperative RTV-to-LTV (mL)	0.16 (−1.07–2.24)	1.14 (−0.168–2.06)	1.000
Postoperative RTV (mL)	7.74 (7.03–8.71)	4.5 (3.66–6.65)	0.147
Postoperative LTV (mL)	6.5 (4.39–8.71)	4.48 (4.39–5.73)	0.286
Postoperative RTV-to-LTV (mL)	0.2 (−0.56–1.24)	−0.46 (−1.29–0.11)	0.364
Change in RTV (%)	38% (5.74–98)	68.8% (4.50–146)	0.933
Change in LTV (%)	34.6% (−21.9–122)	110% (47.1–179)	0.683
Hydrocele	2 (14.3%)	1 (14.3%)	1.000
Calcification	0 (0%)	4 (57.1%)	0.002
Recurrence	3 (21.4%)	1 (14.3%)	0.694
Follow-up (year)	5.5 (2.5; 7)	3 (2; 3)	0.077
Clavien-Madadi Classification			0.343
1	2 (50%)	4 (80%)	
3B	2 (50%)	1 (20%)	

RTV: right testicular volume; LTV: left testicular volume; RTV-to-LTV: difference in right and left testicular volume.

**Table 3:** Comparison according to arterial sparing surgery.

	No Arterial Sparing (n = 17)	Arterial Sparing (n = 4)	*p*-Value
Age	15.5 (13.5–17)	15 (14–15.5)	0.711
Side (left)	17 (100%)	4 (100%)	1.000
Grade 3 varicocele	17 (100%)	4 (100%)	1.000
Preoperative RTV (mL)	6.8 (3.6–7.5)	5.6 (3.3–6.6)	0.559
Preoperative LTV (mL)	6.2 (4.6–7.92)	3.12 (2.08–3.33)	0.064
Preoperative RTV-to-LTV (mL)	0.2 (−1.20–2.05)	2.5 (1.17–3.27)	0.171
Postoperative RTV (mL)	6.84 (3.54–7.43)	8.43 (8.29–8.57)	0.198
Postoperative LTV (mL)	5.67 (4.46–6.83)	8.71 (8.71–8.71)	0.170
Postoperative RTV-to-LTV (mL)	0.155 (−0.9–1.26)	−0.28 (−0.42–−0.14)	0.659
Change in RTV (%)	60.8% (3.82–130)	15.2% (15.2–15.2)	1.000
Change in LTV (%)	69.3% (−22.4–129)	147 (147–147)	0.5
Hydrocele	2 (11.8%)	1 (25%)	0.496
Calcification	4 (23.5%)	0 (0%)	0.281
Recurrence	4 (23.5%)	0 (0%)	0.281
Clavien-Madadi Classification			0.134
1	6 (75%)	0 (0%)	
3B	2 (25%)	1 (100%)	

RTV: right testes volume; LTV: left testes volume; RTV-to-LTV: difference in right and left testicular volume.

## Data Availability

The research data used in the study are available from the correspond-ing author upon reasonable request.
